# A Constitutive Equation and Numerical Study on the Tensile Behavior of Reinforcing Steel Under Different Mass Loss Ratios

**DOI:** 10.3390/ma18112640

**Published:** 2025-06-04

**Authors:** Wei Zhang, Zhilin Long, Xiaowei Liu

**Affiliations:** 1 School of Mechanical Engineering and Mechanics, Xiangtan University, Xiangtan 411105, China; 202031000195@smail.xtu.edu.cn (W.Z.); liuxiaowei011@126.com (X.L.); 2 School of Civil Engineering, Xiangtan University, Xiangtan 411105, China

**Keywords:** corroded reinforcing steel, tensile test, constitutive equation, HDD model

## Abstract

This study investigates the mechanical degradation of HRB400 corroded reinforcing steel induced by corrosion and introduces a tailored constitutive model to capture the influence of mass loss ratios. A series of tensile tests were conducted following chloride-driven wet–dry cycles combined with a simulated marine corrosion environment, enabling the quantification of the relationship between mass loss ratios and mechanical performance. A degradation equation based on mass loss ratios was derived and benchmarked against both experimental data and the existing Hooputra’s Ductile Damage (HDD) model. The proposed equation achieved approximately 80% accuracy in predicting strength reduction across varying corrosion levels. A finite element model incorporating the HDD framework was developed to simulate tensile failure, successfully capturing key degradation characteristics, including reduced yield strength, diminished ductility, and a shortened yield plateau. Unlike other models, it maintained high predictive accuracy even under severe corrosion. These findings demonstrate the model’s potential for structural analysis and reinforcement design in corrosion-prone environments.

## 1. Introduction

Reinforcement corrosion is one of the primary causes of durability degradation in reinforced concrete (RC) structures. In severe cases, it can lead to the brittle failure of structural components and even the catastrophic collapse of buildings, posing serious safety risks. Extensive engineering investigations have shown that steel reinforcement in concrete structures located in alternating wet–dry environments is particularly susceptible to corrosion. This issue has become a critical focus in the durability design of coastal concrete structures, as the resulting direct and indirect losses can far exceed initial expectations. Therefore, understanding the mechanical degradation of corroded reinforcing steel under chloride-induced wet–dry cycles has significant practical value. Corrosion affects the mechanical properties of reinforcing steel through three primary mechanisms, (i) reduction in the average cross-sectional area of the steel bar, (ii) formation of corrosion pits leading to stress concentration, and (iii) alteration of the internal lattice structure due to residual internal stress within the steel [1], resulting in changes in its mechanical behavior. Corrosion not only reduces the effective load-bearing area of the reinforcement but also significantly influences key mechanical and deformation properties, such as yield strength, ultimate stress, yield strain, and ultimate strain [2,3,4,5]. Furthermore, corrosion leads to a reduction in the structural load-bearing capacity and energy dissipation ability [6,7,8]. To assess the impact of reinforcement corrosion on structural performance and evaluate the extent of deterioration in load-carrying capacity, it is essential to comprehensively understand the evolution of the physical and mechanical properties of corroded steel. Therefore, an accurate and thorough analysis of the mechanical and deformation behavior of corroded reinforcing steel is crucial for the durability assessment and safety evaluation of existing RC structures.

During the corrosion process of reinforcing steel, two distinct corrosion patterns typically occur: uniform corrosion and localized corrosion. These patterns are associated with different corrosion mechanisms, including the carbonation of the concrete cover (caused by CO_2_ diffusion), the penetration of aggressive agents (such as Cl^−^, O_2_, and moisture), and the influence of stray currents. Among these, chloride-induced corrosion poses the most severe threat. Hydration products in cement, such as calcium hydroxide, establish a highly alkaline environment around the embedded steel reinforcement, forming a passivating iron oxide film that protects the steel from corrosion. However, carbon dioxide from the atmosphere reacts with hydration products in the concrete, producing calcium carbonate—a process known as carbonation. When carbonation extends to the steel surface, the pH drops significantly, leading to a rapid reduction in the protective effect of the concrete, allowing corrosion to begin. The presence of chloride ions further accelerates this process. Although chlorides do not chemically react directly with the concrete matrix, they facilitate the formation of anodic and cathodic regions within the steel, thereby intensifying corrosion. Under chloride-induced attack, the protective layer surrounding the reinforcement deteriorates [9,10,11]. To quantify the impact of corrosion on reinforcing steel, various degradation models have been developed to describe the reduction in effective cross-sectional load-bearing capacity, providing constitutive equations that characterize the mechanical deterioration of corroded steel [12,13].

Although the degradation of aging reinforced concrete structures [14,15,16,17,18,19,20,21] has received considerable attention, the impact of corrosion on the mechanical properties of reinforcing steel remains an active area of research [22]. In particular, no unified constitutive equation based on the corrosion rate has been established. Some studies have focused on probabilistic models [21,22,23,24], while refined modeling approaches [1,24] remain limited. Notably, three-dimensional solid composite material simulations [1] are even less explored.

There are four primary methods for inducing reinforcement corrosion: (1) natural corrosion in aggressive environments, (2) electrochemically accelerated corrosion tests, (3) artificially simulated environmental tests, and (4) the laboratory-based mechanical simulation of corroded specimens. Most studies have employed constant-current accelerated corrosion on either uncorroded or pre-embedded steel reinforcement [5,17,18,21,23,24,25,26,27,28,29]. While localized corrosion has a relatively minor effect on the strength of reinforcing steel, it significantly deteriorates its plasticity [2,25]. The presence of corrosive solutions promotes the preferential dissolution of the martensitic phase [22,30], reducing work-hardening behavior and accelerating plastic deformation, which in turn increases the corrosion rate [30]. Long-term studies have revealed that electrochemical acceleration differs significantly from natural corrosion conditions. In this study, an artificial climate simulation was employed to achieve accelerated corrosion, offering better alignment with real-world corrosion behavior. This method is increasingly recognized as a key approach in the durability assessment of marine steel structures.

To further interpret the degradation behavior under such complex corrosion environments, mathematical modeling becomes essential. Recent advances in corrosion modeling have employed techniques such as cellular automata, finite element methods, and coupled diffusion–reaction equations to capture pit growth dynamics, mass transport, and electrochemical interactions. These approaches not only enable a more accurate simulation of corrosion morphology but also provide quantitative predictions of mechanical property degradation under various corrosion scenarios. Despite these developments, there is still a need to unify empirical observations and numerical results through a generalized, corrosion rate-dependent constitutive model. This study aims to bridge that gap by incorporating corrosion-induced deterioration into a modeling framework that reflects both experimental observations and theoretical principles.

While numerous studies have focused on naturally corroded reinforcement [6,12,22], research on simulating extreme marine corrosion conditions remains limited. Drawing on the findings of [31,32], researchers have systematically summarized the corrosion mechanisms [33] and fundamental principles governing the behavior of steel structures in marine environments. Recent advancements in mathematical modeling strategies, combined with in situ thickness measurements, have enhanced the qualitative understanding of corrosion processes at different locations within marine structures [34]. Building upon a series of experimental investigations on a specific marine engineering project, researchers analyzed the spatial distribution of corrosion products in concrete. The findings contribute to predicting concrete cracking behavior resulting from chloride-induced steel reinforcement corrosion.

This study investigates the mechanical properties of reinforcing steel degraded by corrosion in marine environments, taking into account the inherent variability in these properties [35]. An artificial climate simulation was used to replicate the corrosion process, resulting in uniform corrosion patterns similar to those observed in long-term coastal exposure tests. A novel constitutive equation was proposed to describe the degradation of mechanical properties as a function of mass loss ratios. Experimental data were used to establish deterioration equations for key mechanical parameters—including elastic modulus, yield strength, ultimate stress, and ultimate strain—under varying mass loss ratios. Finally, a new corrosion rate-dependent degradation equation was developed, demonstrating its applicability in refined finite element modeling. A comparative simulation was performed to support damage and fracture assessment in structural performance evaluations.

## 2. Experimental Program

### 2.1. Specimen Design

**Figure 1:**
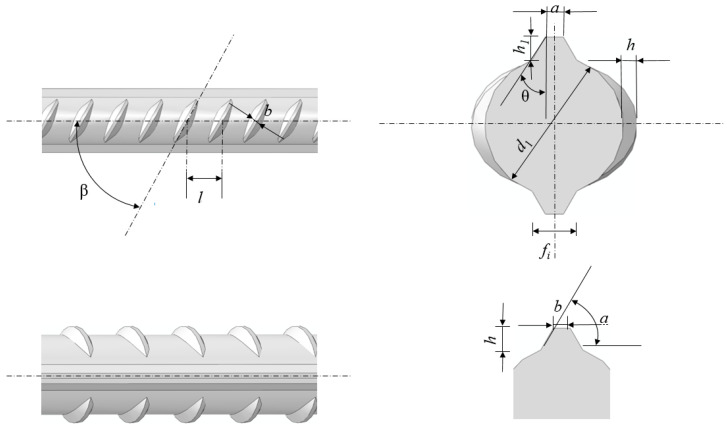
Schematic of reinforcing steel.

HRB400 steel bars with different mass loss ratios were selected and machined into tensile specimens according to ISO/FDIS 15630-1 [36] standards. The chemical composition and geometric parameters are listed in Table 1 and Table 2. A 3D schematic of the specimen is shown in Figure 1. Preliminary tests and previous studies [37] indicate that reinforcing steel, after corrosion treatment, exhibits nearly uniform corrosion, resembling that observed under natural environmental exposure. In contrast, steel embedded in concrete tends to develop localized pitting corrosion under corrosion conditions [23,38,39,40,41,42]. This localized corrosion pattern is characteristic of chloride-induced degradation in steel-reinforced concrete.

To investigate the effects of corrosion, the specimens were divided into groups subjected to different corrosion durations. Before corrosion, the initial mass and dimensions of each specimen were recorded. After corrosion exposure, surface rust was removed using mechanical brushing and tapping. Final mass measurements were taken, and tensile tests were conducted to assess changes in mechanical properties.

Here, *d*_1_ is the inner diameter, *h* is the transverse rib height, *h*_1_ is the longitudinal rib height, *d* is the nominal diameter, *b* is the transverse rib width, *a* is the longitudinal rib width, and *l* is the rib spacing.

### 2.2. Corrosion Process

**Figure 2:**
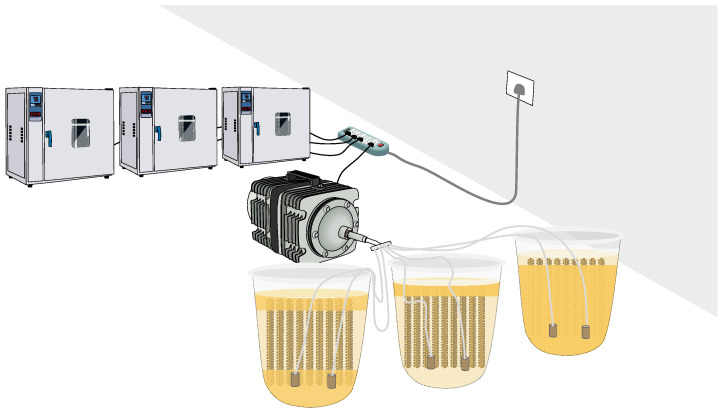
Accelerated corrosion technique.

A constant current was used to simulate seawater corrosion under controlled conditions. A large constant-temperature chamber was employed to accelerate corrosion through heating, dry–wet cycling, and oxygen pumping. As shown in Figure 2, the selected salinities were 0‰, 35‰, and 45‰, with drying temperatures set at 24 °C, 46 °C, and 68 °C. The dry–wet ratios were 5:19, 20:4, and 22:2. The corrosion period ranged from 2 months to 2 years.

At different corrosion intervals, the mass of the specimens was measured using a high-precision electronic balance. After corrosion, the specimens were cleaned by mechanical brushing, and the mass loss was calculated. The corrosion depth and uniformity were also assessed, with the corrosion morphology primarily showing uniform characteristics.

**Figure 3:**
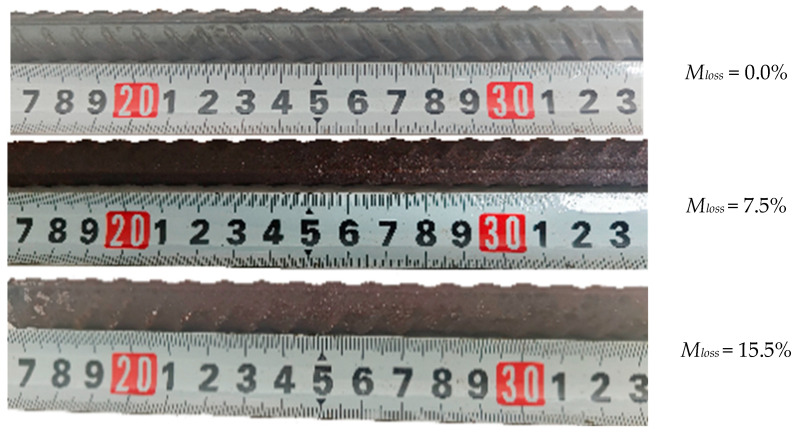
Uniform corrosion on the artificially damaged specimen.

In Equation (1), the effective mass loss was defined by measuring the mass before and after corrosion (*m*_0_ and *m*_1_) [22]. When corrosion rates are low or corrosion is relatively uniform, the cross-sectional area loss of the steel reinforcement is approximately equal to the mass loss rate. Given the ideal control of the corrosion environment in this experiment, the results of the samples tend toward uniform corrosion.

(1) $$M_{\mathrm{loss}}=\frac{m_{0}-m_{1}}{m_{0}}\times 100\%$$

where *M_loss_* is the actual mass loss ratio determined through the experiment, *m*_0_ is the initial mass of the rebar specimen, and *m*_1_ is the mass of the rebar after corrosion. A partially uniformly rusted rebar specimen is shown in Figure 3.

### 2.3. Loading Scheme of the Artificially Corroded Bar Reinforcement

**Figure 4:**
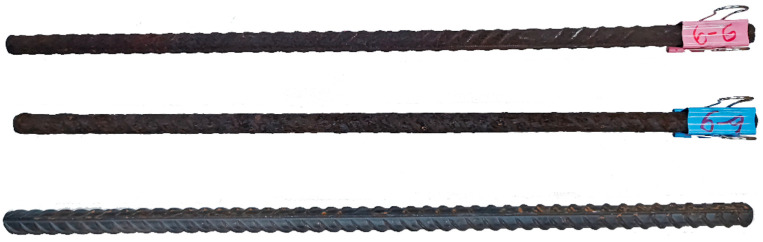
Some specimens, artificially corroded (6-6 and 6-9), before the tensile test.

**Figure 5:**
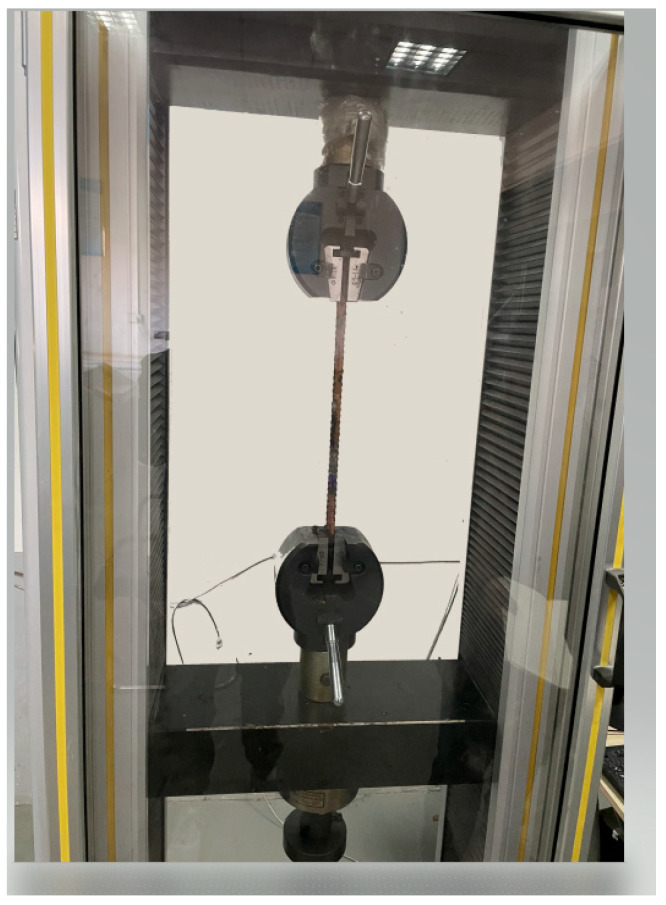
Universal testing machine for 10 tons.

**Figure 6:**
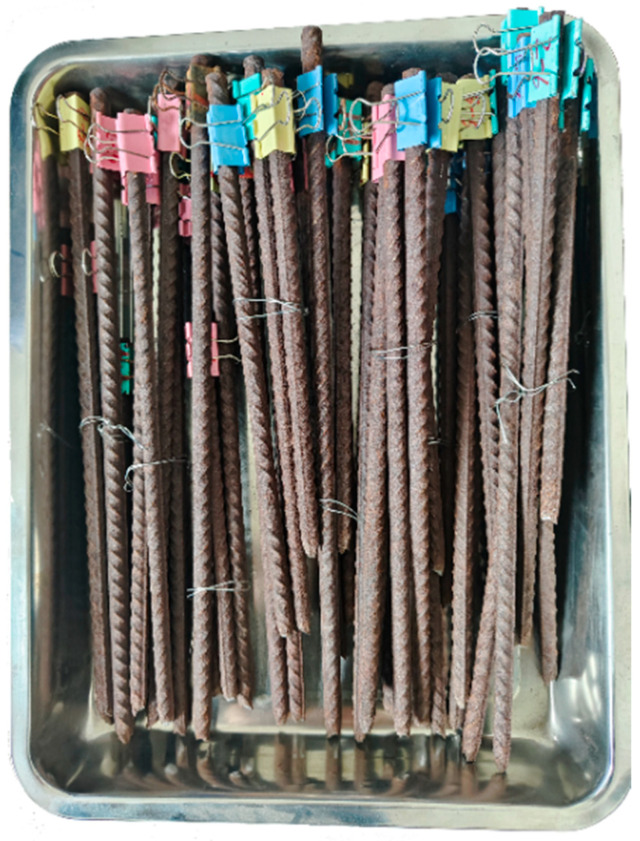
Corroded steel bars.

**Figure 7:**
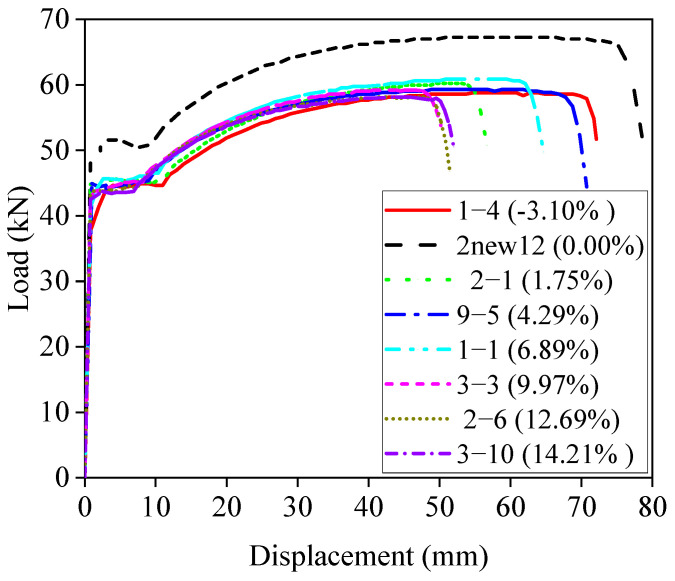
Load–displacement curves.

In this paper, steel bars with a nominal diameter of 12 mm were corroded (some full-length bars are shown in Figure 4), tensile damage experiments were conducted (Figure 5 and Figure 6), and the displacement–load curves were measured, as shown in Figure 7. A total of 64 specimens possessing 61 different degrees of corrosion were evaluated for mass loss ratios based on Equation (1).

After the corrosion process, the corroded steel bars were subjected to tensile tests according to ISO/FDIS 15630-1 [36]. Depending on the bar diameter, an electro-hydraulic servo universal testing machine (WDW-100, China Changsha, Lihuan Testing Instrument Equipment Co., Ltd.) with a 10-ton capacity was used to perform monotonic tensile tests on the specimens. The elastic moduli at both ends of each bar were measured prior to testing using an electronic extensometer with a gauge length of 100 mm. The extensometer was removed before the fracture to prevent damage to the device. All tensile tests were conducted at a constant displacement rate of 10 mm/min at room temperature, and the total length of each specimen was 400 mm. Load–displacement curves under different mass loss ratios for variously labeled specimens were obtained experimentally (Figure 7) to evaluate the yield strength, ultimate stress, and elongation of corroded rebars.

**Figure 8:**
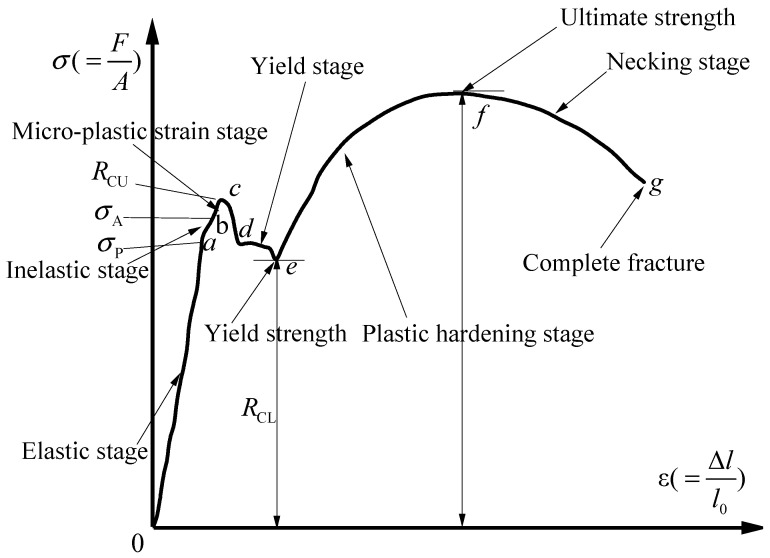
Definition of the relevant physical quantity on the stress–strain curve.

The tensile response was further characterized by the stress–strain relationships (Figure 8), which defined key mechanical parameters for subsequent analyses, including yield strength and ultimate stress. Specifically, stress values were calculated based on both the nominal cross-sectional area of the reinforcement (engineering stress) and the reduced cross-sectional area after corrosion (effective stress). In both cases, the mechanical properties showed a clear degradation trend with increasing mass loss ratios, which will be comparatively analyzed in the following sections.

According to Eurocode (EN 1990:2002 [43]), the design value of any geometrical characteristic in reinforced concrete structures is defined as the nominal value. Therefore, when analyzing an existing structure or component, the nominal cross-sectional dimensions should be used rather than those altered by corrosion. Accordingly, the nominal cross-section is adopted in the subsequent finite element simulation analyses.

## 3. Experimental Results and Analysis

### 3.1. Surface Topography

**Figure 9:**
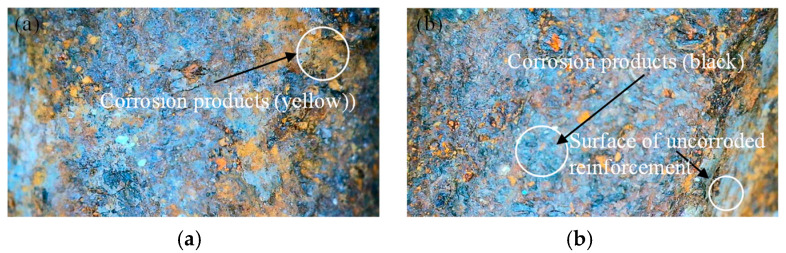
Reinforcement corrosion morphology. (**a**) Corrosion products are yellow in color. (**b**) Corrosion products are black in color.

Changes in the surface morphology of the specimens exposed to the marine environment were observed using scanning electron microscopy (SEM). Due to the large number of specimens, only the selected single-sided samples are presented (see Figure 9). The images show the presence of yellow and black corrosion products.

Surface morphology analysis revealed the following:

(a) In the early stages of corrosion, the localized areas of the specimen exhibited severe corrosion. Numerous pinholes appeared on the surface, with corrosion propagating in the vertical direction. This was clearly visible to the naked eye after surface cleaning.

(b) As the corrosion duration increased, the corrosion extended across the entire surface. With the gradual interconnection of pinholes, larger and deeper pits formed. Various types of pitting morphologies were observed, including U-shaped, elliptical, and conical pits [44].

(c) In the advanced stages of corrosion, pitting continued to develop and expand, some primary pits gave rise to secondary pits, and some steel bar surfaces spalled as rusting progressed toward uniform corrosion.

Yellow corrosion products are typically ferric hydroxide (Fe(OH)_3_) or hydrated ferric oxide (FeO(OH)•nH_2_O), which form as rust in oxygen-rich and humid environments. These products appear bright yellow or reddish-brown. Black corrosion products may consist of magnetite (Fe_3_O_4_) or ferrous hydroxide (Fe(OH)_2_), generally forming under oxygen-deficient or -reducing conditions. These are denser corrosion products that may offer temporary protection to the underlying metal. Therefore, the different colors not only indicate variations in the chemical composition of corrosion products but also reflect spatial heterogeneity in the corrosion process and differences in the local electrochemical environment.

### 3.2. Tensile Failure Morphology

**Figure 10:**
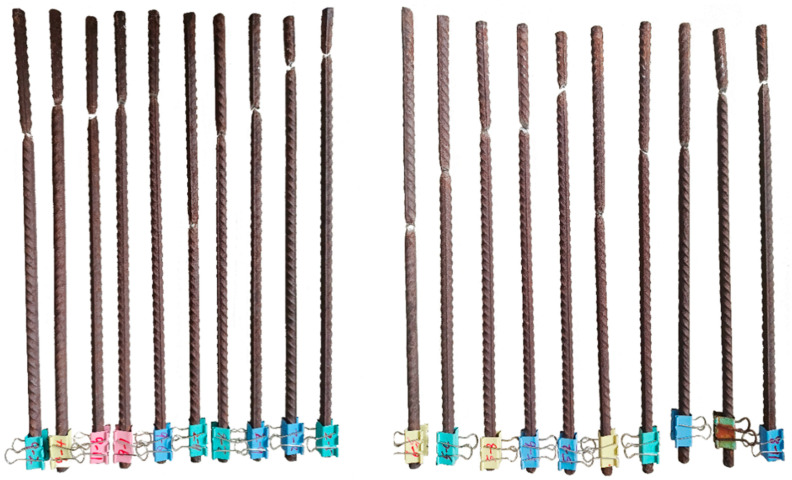
Tensile failure modes of corroded rebars.

**Figure 11:**
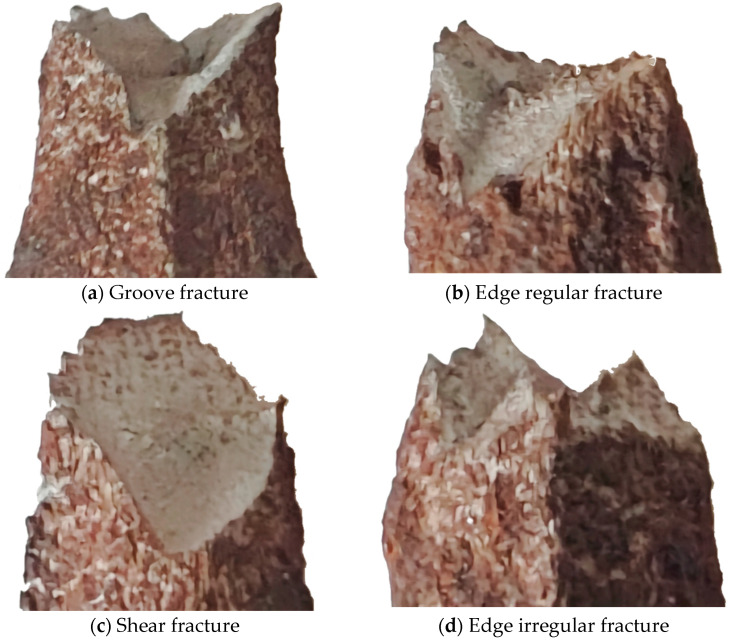
Fracture morphology.

The damage morphology of the corroded bars is shown in Figure 10 and Figure 11 (Figure 10 shows the overall corrosion morphology and Figure 11 shows the port corrosion morphology). From the experiment, it can be seen that (a) all specimens loaded to fracture still exhibit the necking phenomenon; (b) the surface of the steel bar becomes rough due to corrosion, and the fracture locations are mainly concentrated near the two ends, with fewer occurring near the middle, as partially shown in Figure 10. Observe and compare the fracture morphology as shown in Figure 11: the fracture morphology of the specimen without corrosion or without obvious corrosion is basically a groove type, the fracture edge is a regular arc, a cup and cone fracture, in line with the toughness fracture characteristics (Figure 11a,b). The fracture morphology of the rusted specimen with obvious pitting pits on the surface is a shear type or irregular edge. The shear fracture was caused by severe corrosion, as shown in Figure 11c. In contrast, specimens with obvious rust morphology have irregular fracture paths due to stress concentration caused by pitting pits, as shown in Figure 11c,d. Larger pits are detected where fractures occur due to surface damage and the uniformity of material properties caused by corrosion, as well as the effects of the weak cross-section of the pitting and stress concentration. At high stress levels, pitting on the specimen surface may sprout and expand cracks at a faster rate [12].

### 3.3. Tensile Stress–Strain Curves at Different Mass Loss Ratios

**Figure 12:**
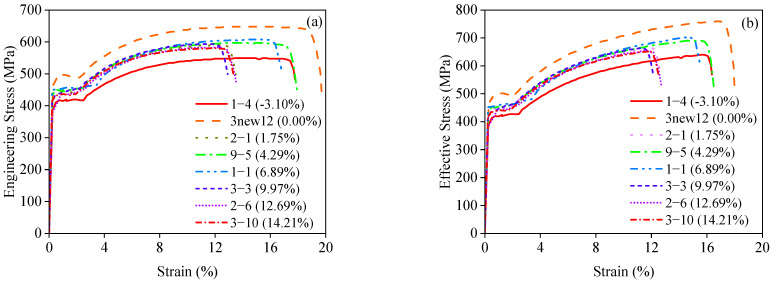
Stress–strain relationships for the corroded reinforcing steels: (**a**) engineering stresses; (**b**) effective stresses.

Figure 12 presents the stress–strain curves of some of the specimens. Some of the bars exhibit negative mass loss ratios, indicating that the bars are in the early stages of corrosion and the corrosion products strongly adhere to the bars, resulting in an apparent mass gain. The results show that the yield strength, ultimate stress, and ductility of the bars decrease with increasing mass loss ratios, although the degree of degradation is relatively limited, where Figure 12a shows the engineering stress–strain curve and Figure 12b shows the true stress–strain curve. From Figure 12, it can be seen that the stress–strain curves of the reinforcement bars with different mass loss ratios reflect different decay patterns as a function of mass loss.

### 3.4. Effects of Mass Loss Ratios on Mechanical Properties

In previous studies [4,22], the nominal stress was calculated using the initial cross-sectional area, and the value was much smaller than the actual value, affecting the degradation of mechanical properties caused by localized pitting corrosion. Since the corrosion is dominated by uniform corrosion [22], this paper adopts the measured cross-sectional area method to calculate the reasonable nominal stress, which is closer to the real stress.

According to the test results, the mechanical properties of the HRB400 rebar specimens are shown in Table 3. Table 3 shows the mass loss ratios *M_los_*_s_, the lower yield limit *F_el_*, the ultimate load-carrying capacity *F_m_*, the yield strength *f_y_*, the ultimate stress *f_u_*, the fracture strength *f_br_*, the flexural strength ratios *f*_y_/*f*_u_, the relative yield strength *k_y_*, the relative ultimate stress *k_u_*, and the elongation after break *δ*. The data in Table 3 show, as a whole, that the mechanical properties of the reinforcement bars decrease with the increase in the corrosion time.

The decrease in the mechanical properties of the material is mainly due to the damage to the material surface caused by corrosion. On the one hand, the cross-section is reduced; on the other hand, there is a stress concentration in the pitting pits. The stress concentration increases the local stress level, leading to yield advancement, which in turn accelerates the emergence of a plastic zone on the surface, resulting in non-uniform plastic deformation in the center of the material and the surface layer with a larger deformation amplitude, and the higher the stress concentration coefficient is, the weaker the plastic deformation capacity is. Therefore, the decrease in plasticity may be due to the pitting craters on the surface of the specimen, which cause the cracks in the material to sprout and expand faster at high stress levels.

**Figure 13:**
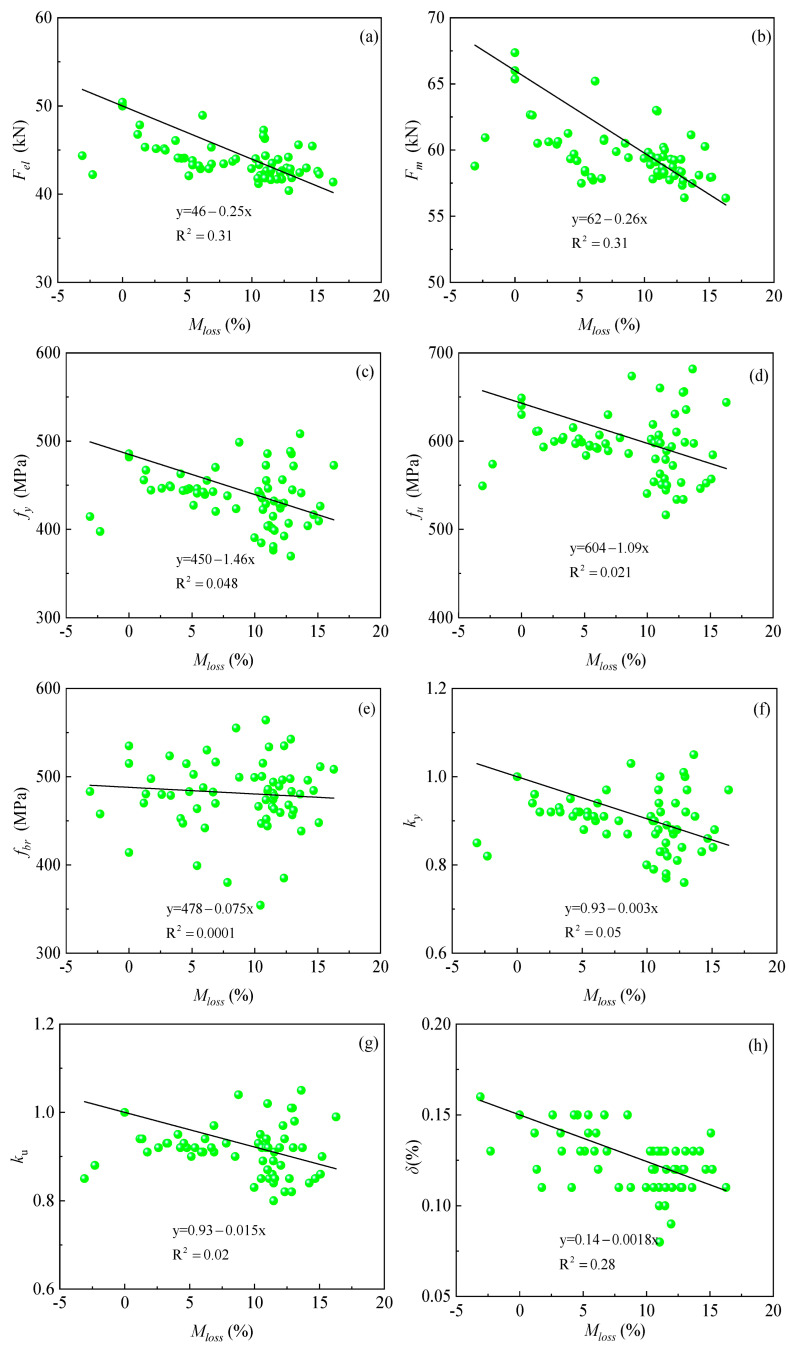
Plot of mass loss ratios vs. mechanical parameters of each rebar: (**a**) *F_el_* and *M_loss_*; (**b**) *F_m_* and *M_loss_*; (**c**) *f*_y_ and *M_loss_*; (**d**) *f*_u_ and *M_loss_*; (**e**) *f_br_* and *M_loss_*; (**f**) *k_y_* and *M_loss_*; (**g**) *k_u_* and *M_loss_*; (**h**) *δ* and *M_loss_*.

As shown in Figure 13 below, the lower yield force limit *F_el_*, ultimate load-carrying capacity *F_m_*, yield strength *f_y_*, ultimate stress *f_u_*, fracture strength *f_br_*, relative yield strength *k_y_*, relative ultimate stress *k_u_*, and elongation after break *δ* show an overall decreasing trend as the mass loss ratio increases. The linear fitting equations and coefficients of determination (R^2^) for the mass loss ratios and mechanical parameters are given in Figure 13a–h. From the figure, it can be seen that the mechanical property parameters of the corroded steel bars show a large dispersion, a phenomenon that is consistent with the findings in the related literature [6,23,37]. Among them, the regression formula of yield strength *f_y_*, ultimate stress *f_u_*, and fracture strength *f_br_* will provide a reference basis for the calculation of the intrinsic equation based on the mass loss ratios; the calculation of elongation after break *δ* provides an experimental reference basis for the accuracy of finite element simulation results.

## 4. Mechanical Properties Degradation Model

### 4.1. Constitutive Model

**Figure 14:**
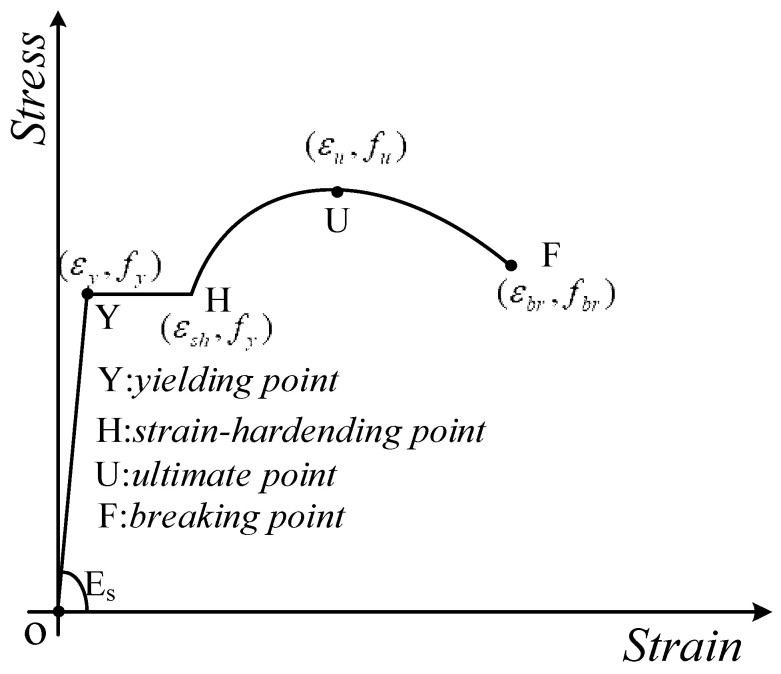
Constitutive model.

As shown in Figure 14, a four-stage principal model [12] is applied to the monotonic loading curve of the reinforcement: the first stage is the elastic stage (OY), the second is the yielding stage (Y-H), the third is the strengthening stage (H-U), and the fourth load is the ultimate load before the necking stage of fracture (U-F), which is expressed mathematically as shown in Equation (2).

(2) $$\sigma =\left\{\begin{array}{cc} E_{s}\varepsilon & \left(\varepsilon <\varepsilon_{y}\right)\\ f_{y} & \left(\varepsilon_{y}\leq \varepsilon <\varepsilon_{sh}\right)\\ f_{u}+\left(f_{y}-f_{u}\right){\left(\frac{\varepsilon_{u}-\varepsilon }{\varepsilon_{u}-\varepsilon_{sh}}\right)}^{P_{1}} & \left(\varepsilon_{sh}\leq \varepsilon <\varepsilon_{u}\right)\\ f_{u}-\left(f_{u}-f_{br}\right){\left(\frac{\varepsilon -\varepsilon_{u}}{\varepsilon_{br}-\varepsilon_{u}}\right)}^{P_{2}} & \left(\varepsilon_{u}\leq \varepsilon <\varepsilon_{br}\right) \end{array}\right.$$

where *E_s_*: modulus of elasticity, *f_y_*: yield stress, *f_u_*: ultimate stress, *f_br_*: fracture stress, *ε_y_*: yield strain, *ε_u_*: ultimate strain, *ε_sh_*: hardening point strain, *ε_br_*: fracture strain, *P*_1_: the shape parameter of the ascending section, and *P*_2_: the parameters of the descending section.

### 4.2. Comparison Between Experimental Model and Computational Model

**Figure 15:**
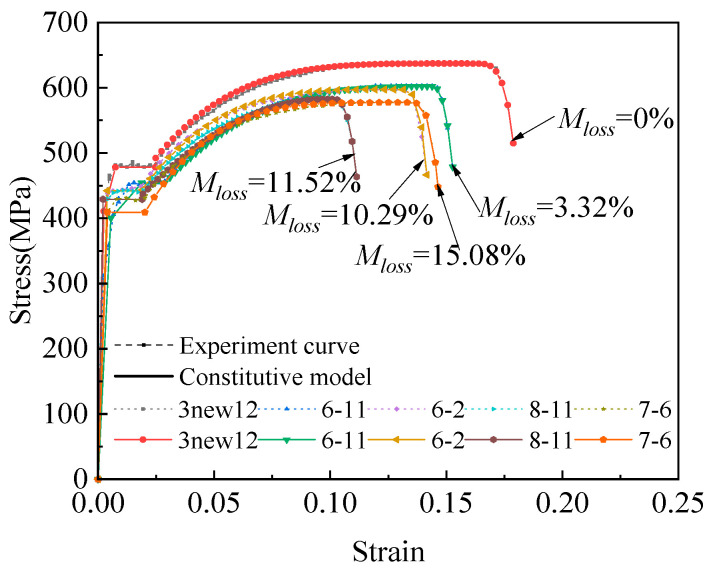
Comparison chart of experiment curve and constitutive model.

Some experimental data (specimens 3new12, 6-11, 6-2, 8-11, and 7-6) are compared with the intrinsic model, as shown in Figure 15 (where the dashed line represents the experimental data and the solid line represents the intrinsic model). The model parameters for all the specimens are presented in Table 4. From Figure 15 and Table 4, it can be observed that the fitting results of the intrinsic model are excellent, with confidence intervals within 95%. The mass loss ratios are within 16.28%. The stress–strain curve exhibits a clear yield plateau. As the corrosion degree increases, the yield plateau shortens, and the yield strain and ultimate strain of the corroded rebar decrease. The stress concentration caused by pitting pits increases the local stress level, leading to yield initiation. This, in turn, accelerates the formation of a plastic zone on the surface, intensifies the inhomogeneous plastic deformation in both the center and surface layers of the material, and shortens the yield plateau. Moreover, the larger the stress concentration coefficient, the shorter the yield plateau becomes.

**Figure 16:**
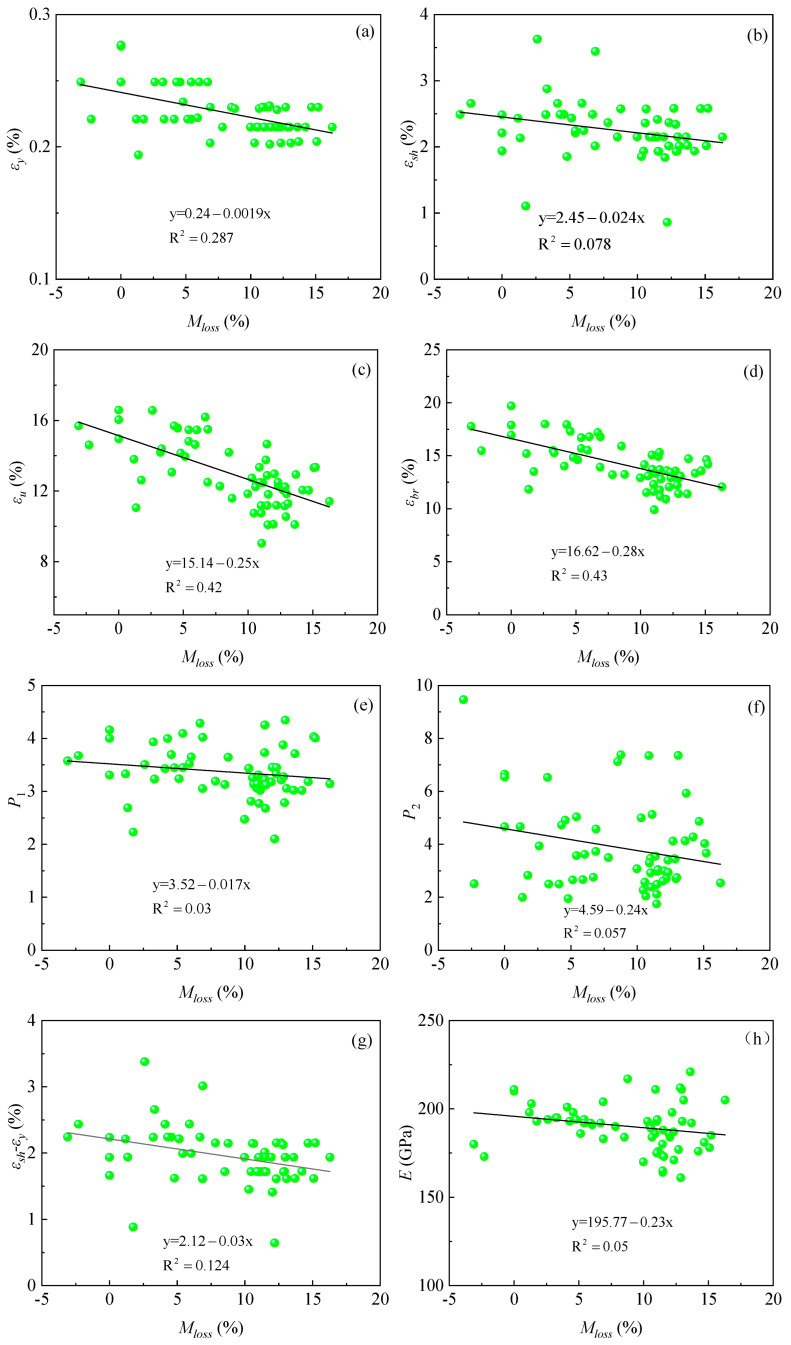
Plot of reinforcing steel constitutive parameters versus mass loss ratios: (**a**) *ε_y_* and *M_loss_*; (**b**) *ε_u_* and *M_loss_*; (**c**) *ε_sh_* and *M_loss_*; (**d**) *ε_br_* and *M_loss_*; (**e**) *P*_1_ and *M_loss_*; (**f**) *P*_2_ and *M_loss_*; (**g**) *ε_sh_*-*ε_y_* and *M_loss_*; (**h**) *E* and *M_loss_*.

In this paper, the formula is fitted to all the experimental data to obtain the model parameters based on the mass loss ratios. The parameters of the intrinsic model for both unrusted and rusted steel bars are provided in Table 4. Due to the large data discretization, visualizing the relationship between the mass loss ratios and each model parameter directly from Table 4 is challenging. This is illustrated in Figure 16 below.

As shown in Figure 16, the yield strain *ε_y_*, the hardening point strain *ε_sh_*, the ultimate strain *ε_u_*, the fracture strain *ε_br_*, the shape parameter of the ascending section *P*_1_, the descending section parameter *P*_2_, the yield plateau *ε_sh_*-*ε_y_*, and the modulus of elasticity *E_s_* (the subscript *s* is the abbreviation of steel, which is denoted as *E* in the following) show an overall decreasing tendency with the increase in the mass loss ratios. With the increase in the mass loss ratios, the yield platform shortens, based on the experimental data processing aspects (rather than on the different mass loss ratios, intuitively verified by the stress–strain curve of the yield platform), which again overall verified that the higher the mass loss ratios, the shorter the yield platform. Among them, the regression formulae of parameters *ε_y_*, *ε_sh_*, *ε_u_*, *ε_br_*, *P*_1_, *P*_2_, and *E* will provide a reference for the calculation of the intrinsic model based on the mass loss ratios.

### 4.3. Mechanical Degradation Model

Combining Section 3.2 and Section 4.2, the relationship between the mass loss ratios and the 10 ontological parameters was derived, and the fitted equations are shown in Figure 13 and Figure 16 above, which can be used as a unified ontological equation related to the mass loss ratios. Overall, the parameters *f_y_*, *f_u_*, *f_br_*, *ε_y_*, *ε_u_*, *ε_sh_*, *ε_br_*, *P*_1_, *P*_2_, and *E* decrease as a whole with the increase in the mass loss ratios (*M_loss_*). In the following, the new intrinsic equation of mass loss ratios is proposed. Firstly, the values of parameters *f_y_*, *f_u_*, *f_br_*, *ε_y_*, *ε_u_*, *ε_sh_*, *ε_br_*, *P*_1_, *P*_2_, and *E* are evaluated by different mass loss ratios (*M_loss_*), and the obtained values are brought into Equation (2). Then, the curve expressions for each segment are calculated, and finally, the intrinsic equations (Equation (3)) are calculated for different mass loss ratios by using the specific expressions.

(3) $$\sigma =\left\{\begin{array}{cc} (195.77-0.23M_{loss})\varepsilon & \left(\varepsilon <0.24-0.0019M_{loss}\right)\\ 450-1.46M_{loss} & \left(0.24-0.0019M_{loss}\leq \varepsilon <2.45-0.024M_{loss}\right)\\ 604-1.09M_{loss}-\left(0.37M_{loss}+154\right){\left(\frac{(15.14-0.25M_{loss})-\varepsilon }{12.69-0.226M_{loss}}\right)}^{3.52-0.017M_{loss}} & \left(2.45-0.024M_{loss}\leq \varepsilon <15.14-0.25M_{loss}\right)\\ 604-1.09M_{loss}-\left(126-1.015M_{loss}\right){\left(\frac{\varepsilon -(15.14-0.25M_{loss})}{1.48-0.03M_{loss}}\right)}^{4.59-0.24M_{loss}} & \left(15.14-0.25M_{loss}\leq \varepsilon <16.62-0.28M_{loss}\right) \end{array}\right.$$

## 5. Numerical Simulation

Considering that the elastic–plastic model of the steel bar can be implemented in finite element software, the necking and fracture behavior needs to be simulated using the built-in Hooputra Ductile Damage (HDD) model [45]. This combined approach allows the simulation results to better match the constitutive equation based on the mass loss ratios. It shows that the eigenstructure equation for the decay has high applicability.

### 5.1. HDD Guidelines

(4) $$\eta =\frac{\sigma_{m}}{\sigma_{eq}}=\frac{(\sigma_{1}+\sigma_{2}+\sigma_{3})/3}{\sqrt{{\left(\sigma_{1}-\sigma_{2}\right)}^{2}+{\left(\sigma_{2}-\sigma_{3}\right)}^{2}+{\left(\sigma_{3}-\sigma_{1}\right)}^{2}}/\sqrt{2}}$$

where σ_1_, σ_2,_ and σ_3_ are the principal stress.

Hydrostatic stress:

(5) $$\sigma_{m}=(\sigma_{1}+\sigma_{2}+\sigma_{3})/3$$

Equivalent stress (Mises stress):

(6) $$\sigma_{eq}=\sqrt{{\left(\sigma_{1}-\sigma_{2}\right)}^{2}+{\left(\sigma_{2}-\sigma_{3}\right)}^{2}+{\left(\sigma_{3}-\sigma_{1}\right)}^{2}}/\sqrt{2}$$

For axial stretching, the principal stresses in both directions are 0. Because of the uniaxial tensile test, the triaxiality of the stresses is taken to be 0.33, since quasi-static stretching can be regarded as a strain rate equal to zero.

$\varepsilon_{eq}^{**}$, $\sigma_{m}$, $\sigma_{\mathrm{eq}}$, and η are the equivalent plastic strain at fracture (fracture strain), the average stress, the von Mises equivalent stress, and the stress triaxiality, respectively (Abaqus Documentation). It is a monotonically increasing state variable with increasing plastic deformation, which is defined as follows.

(7) $$D=\int_{0}^{\varepsilon_{eq}^{**}}\frac{d\varepsilon_{eq}}{\varepsilon_{eq}^{**}(\eta )}=1$$

**Figure 17:**
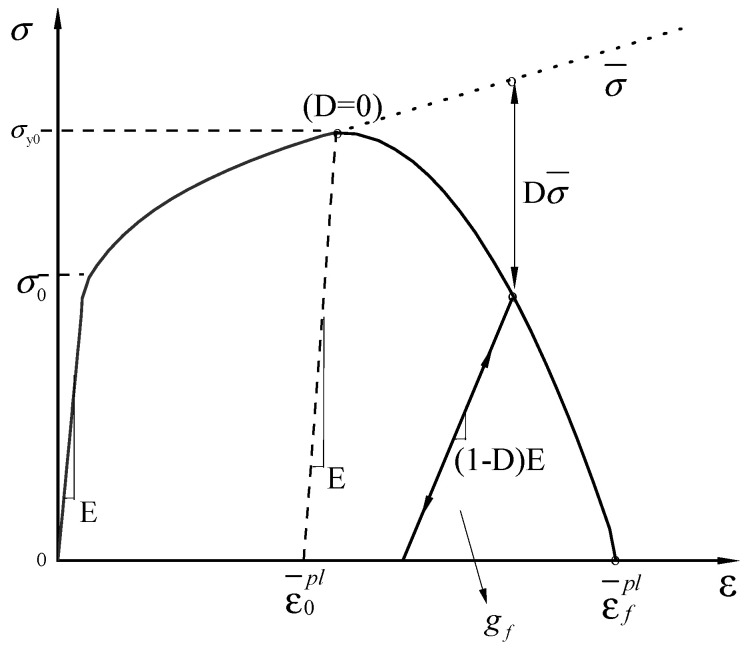
HDD guidelines.

In the above integral fracture criterion, *D* denotes the damage variable, which changes from 0 (original material) to 1 (material fracture) and increases monotonically with the equivalent plastic strain at each increment during the analysis, as shown in the following equation.

(8) $$\frac{\Delta \varepsilon_{eq}}{\varepsilon_{eq}^{**}\left(\eta \right)}\geq 0$$

(9) $$\sigma =(1-D)\bar{\sigma }$$

where $\bar{\sigma }$ is the effective (undamaged) tensile stress at the current incremental step of the calculation, and σ is the stress considering the damage factor, as shown in Figure 17.

Failure displacement: In finite element simulation, once damage softening is considered, the problem of strain localization is encountered (local cell damage enters the softening segment while other cells enter the unloading state), which results in softening developing only in the local cell, and the smaller the cell size, the smaller the energy dissipation of softening damage, leading to mesh size dependence. In order to solve this problem, the fracture energy or the failure displacement is generally used as the material parameter, while the feature length (cell size) is introduced.

*ε* is the total strain, ${\bar{\varepsilon }}^{pl}$ is the equivalent plastic strain, *σ*_0_ is the initial yield stress, *σ*_*y*0_ is the yield stress at damage initiation, ${{\bar{\varepsilon }}_{0}}^{pl}$ is the plastic strain at the onset of damage, ${{\bar{\varepsilon }}_{0}}^{pl}$ is the plastic strain at failure, *(1−D)E* is the reduced stiffness after, and *D* $\bar{\sigma }$ is the stress loss due to damage.

Fracture energy:

(10) $$G_{f}=\int_{{\bar{\varepsilon }}_{0}^{pl}}^{{\bar{\varepsilon }}_{f}^{pl}}L\sigma_{y}d{\bar{\varepsilon }}^{pl}=\int_{0}^{{\bar{u}}_{f}^{pl}}\sigma_{y}d{\bar{u}}^{pl}$$

(11) $$G_{f}=Lg_{f}$$

Failure displacement:

(12) $${\bar{u}}_{f}^{pl}=L({\bar{\varepsilon }}_{f}^{pl}-{\bar{\varepsilon }}_{0}^{pl})$$

**Figure 18:**
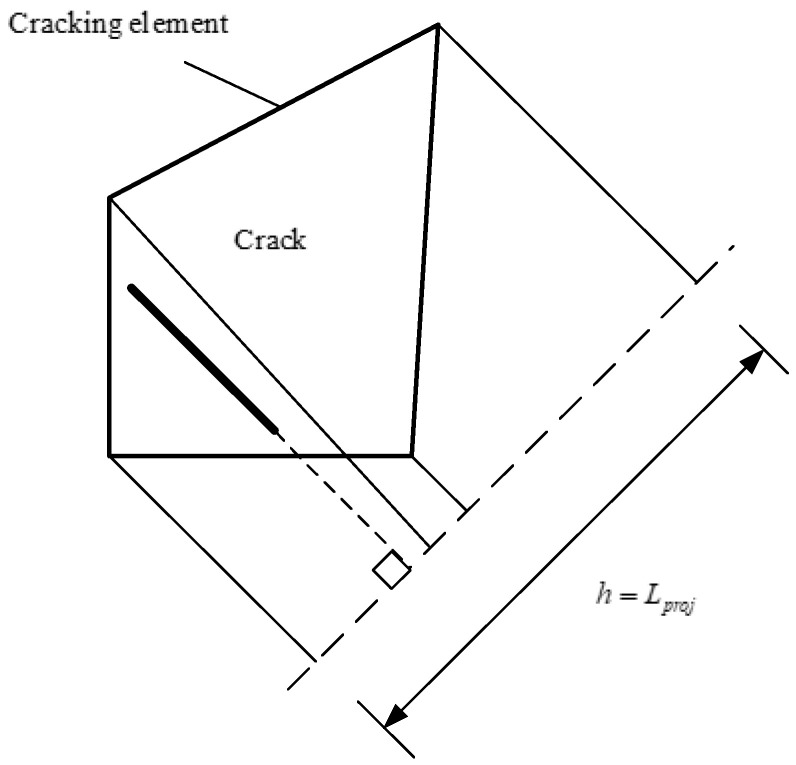
Definition of feature length in HDD.

The characteristic length, *L*, is usually taken as the cell size in the perpendicular crack direction, as shown in Figure 18 above (*L* is equivalent to *L_proj_*).

The definition of the characteristic length depends on the cell geometry and order: for first-order cells, it is the typical length of the line through the cell, whereas for second-order cells, it is half of the typical length. For beams and trusses, the characteristic length is considered along the member axis. Table 5 shows the mechanical properties of the HRB400 bars labeled 2new12, which are used for HDD parameter input.

### 5.2. Finite Element Refinement Modeling and Analysis

#### 5.2.1. Model Establishment and Grid Division

**Figure 19:**
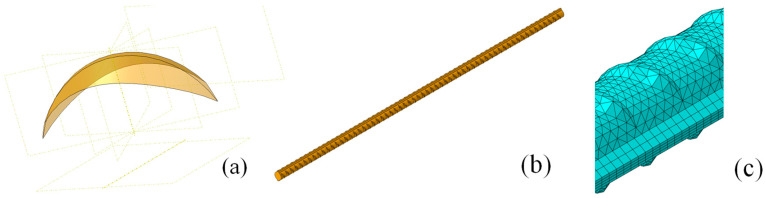
Three-dimensional modeling of rebar. (**a**) Crescent lines on the surface of steel bars. (**b**) Refined rebar modeling. (**c**) Meshing.

The Python 2.7 and finite element interface articulation is very powerful. It allows the input of parametric model parameters, which are then imported into the finite element software to generate a refined model with ribs, as shown in Figure 19 below. Through Boolean operations, the shells are converted to solids, enabling the creation of complex 3D models. The model is 400 mm long with a nominal diameter of 12 mm and is constructed according to the specific parameters in Table 2.

**Figure 20:**
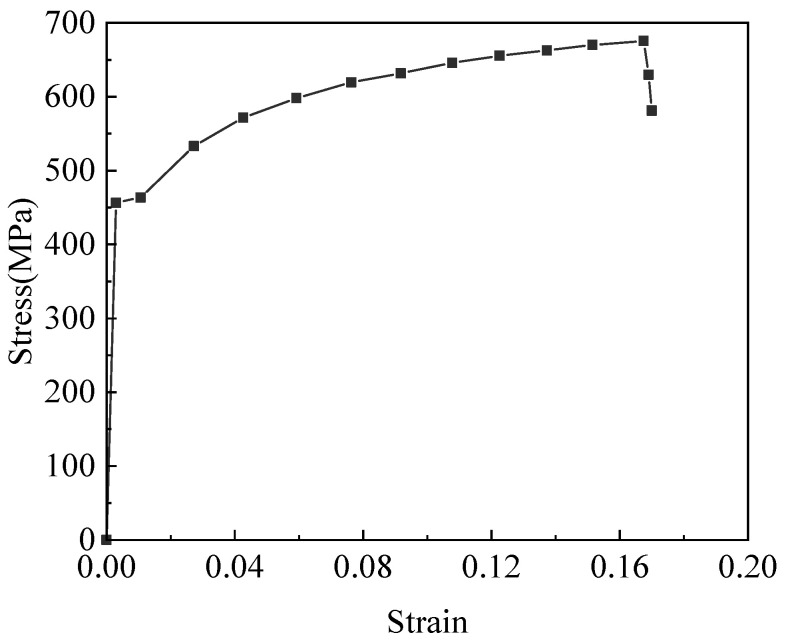
Stress–strain curve of model.

In this paper, the regular part of the 3D model is meshed using C3D8R elements, while the ribbed sections are meshed with C3D4 elements, as shown in Figure 20 below. The mesh, generated through free meshing, has a typical element size of 1.25, with tetrahedral elements predominating. To balance accuracy and computational efficiency, and considering hardware limitations, the total number of elements is controlled between 100,000 and 300,000. In this study, the final mesh consists of approximately 210,000 elements.

#### 5.2.2. Loading Application and Boundary Conditions

The experimental results show that the corrosion-free reinforcement has a modulus of elasticity of *E_s_* = 2.1 × 10^5^ MPa and a Poisson’s ratio of ν = 0.28. Yield, hardening, and ultimate points are found from the experimental nominal stress–strain curves of 2new12, and then a few smooth points are found between the nodes. The actual stress–strain relationship should be entered in the finite element. True stress = Engineering stress × (1 + Engineering strain) and True strain = ln (1 + Engineering strain) (Figure 20).

A multilinear kinematic hardening model and a displacement-controlled loading method are used. Fixed boundary conditions are applied at a position 50 mm from one end of the model to simplify the calculation and facilitate convergence. Displacement loading is applied at a position 50 mm from the other end.

#### 5.2.3. Stress Distribution Analysis

**Figure 21:**
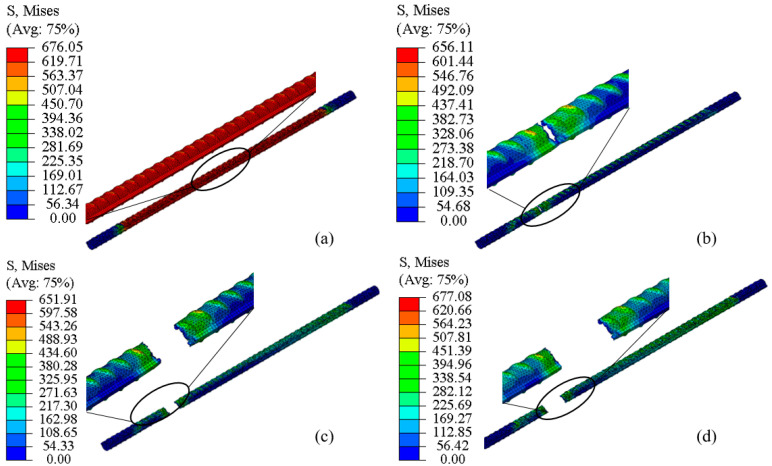
Stress cloud during tensioning of steel bars: (**a**) S.Mises and time = 0.3 s; (**b**) S.Mises and time = 0.4 s; (**c**) S.Mises and time = 0.5 s; (**d**) S.Mises and time = 0.6 s.

As shown in Figure 21 for case 5-3, the stress diagrams at various loading steps indicate that the ribbed geometry of the reinforcement bar leads to surface irregularities and a non-uniform stress distribution. In the elastic deformation stage, the stress of the bar increases linearly, and when it reaches the yielding stage, the stress increases slightly, and the stress is at a maximum when the bar reaches the strengthening stage, which can be seen in Figure 21a; Figure 21b,c indicates the necking stage, and the stress decreases; Figure 21c,d indicates the instant of pulling off, and the stress briefly becomes bigger after the rebound occurs around the pulling off, which even exceeds the value of the stress shown in Figure 21a; from Figure 21a, it can be seen that the simulated location of the bar pull-off and the actual situation match well (see Figure 10), biased toward both ends.

**Figure 22:**
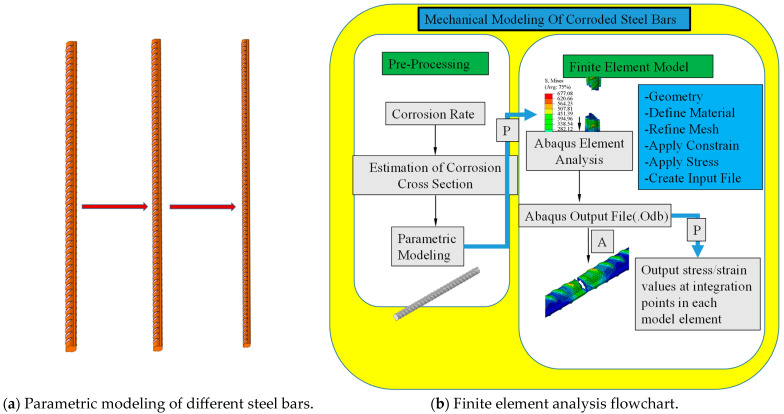
Flowchart of finite element analysis with parametric modeling at different mass loss ratios.

Figure 21a–d illustrate that the refined simulation of the steel bar pull-off process enables a more detailed assessment of the stress distribution within and between the ribs. Notably, following failure, stress in the rib regions tends to dissipate more slowly than in the inter-rib areas. Before pulling off, the overall stress distribution is uniform, and the stress at the clevis end is smaller (fixed end, less deformation); from Figure 21, it can be seen that after pulling off, the stress is larger at the fracture and smaller at other locations. Figure 22 is the finite element analysis flowchart of parametric modeling. The labels “a” and “b” in the figure represent the parametric modeling of the corroded rebar and the finite element analysis workflow, respectively. According to the following process, this paper batch carried out the finite element simulation and analysis of the corroded steel bar model.

#### 5.2.4. Contrastive Analysis of Stress–Strain Relationship

The load corresponding to the displacement is obtained by extracting the total reaction force of the predefined set (Set-1), which is used to generate the load–displacement curve. The load is then divided by the average corroded cross-sectional area of the steel bar to calculate the stress, and the displacement is divided by the original gauge length to obtain the nominal strain, resulting in the nominal stress–strain curve.

**Figure 23:**
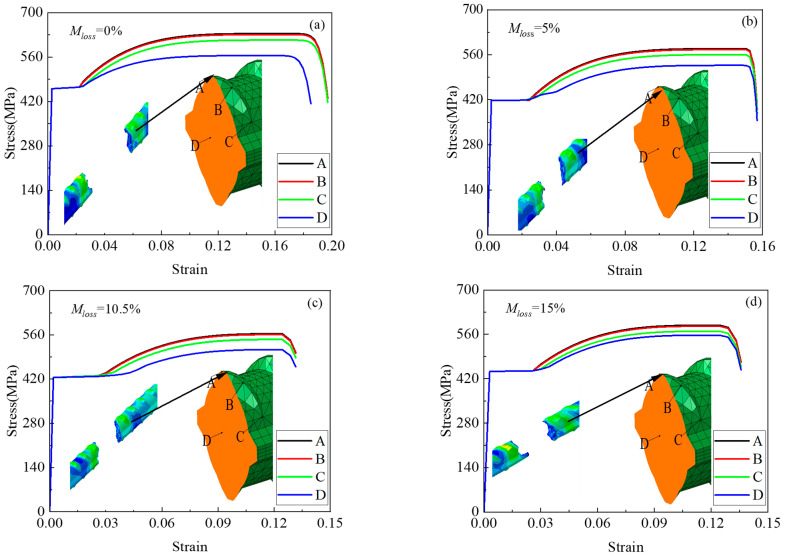
Stress–strain curves of ribbed steel bars at different section locations for different mass loss ratios: (**a**) *M_loss_* = 0%, stress and strain; (**b**) *M_loss_* = 5%, stress and strain; (**c**) *M_los_*_s_ = 10.5%, stress and strain; (**d**) *M_loss_* = 15%, stress and strain.

As shown in Figure 23, for a section near the fracture location, four points (points A, B, and C on the ribbing and point D at the center of the section) were selected at the section and ribbing, respectively. Figure 23a–d show the rebar specimens 2new12, 2-4, 2-11, and 8-6, respectively, which represent different mass loss ratios (0%, 5%, 10.5%, and 15%), as shown in Figure 23 below. From the figure, it can be seen that near the fracture location, the stress at point D in the center of the section is greater than that at point C at the junction of the ribbed and smooth surfaces, and the stress at point A is the smallest. This is completely consistent with the unit deletion in the finite element simulation process: the first unit to be deleted is the first to reach the ultimate stress; in this paper, first, the middle of the rebar yields, and then, the rebar around it yields, and the stress at the ribbing is the smallest, as can be seen from Figure 21. The elastic phase and the yield plateau largely overlap, whereas the plastic phase does not. This suggests that the central region of the section enters the strain-hardening phase earlier than the perimeter, resulting in greater plastic deformation in the middle. The subsequent drop in stress is attributed to the fracture of the rebar—once it breaks, tensile deformation ends, and a portion of the stored elastic energy begins to release, causing the stress to decrease. Meanwhile, other points along the rebar have not yet reached their ultimate stress, but as the rebar as a whole fails, elastic rebound begins, further contributing to the stress reduction.

### 5.3. Models of Stress–Strain Relationship

First, the mass loss ratios are substituted into Equation (3) to obtain *f_y_*, *f_u_*, *f_br_*, *ε_y_*, *ε_u_*, *ε_sh_*, *ε_br_*, *P*_1_, *P*_2_, and ***E***, As shown in Table 6. The new constitutive equations based on different mass loss ratios are calculated, and the following simulations are carried out to validate the constitutive equations based on different mass loss ratios with the engineering stress–strain in the experimental data and the true stress–strain in the HDD model.

The HDD model in this paper is based on the model under different mass loss ratios, which are able to simulate the method of material fracture simulation. The HDD model is able to realize the deletion of the unit, perform the simulation of rebar tensile fracture, parametrically model the rebar for different mass loss ratios, and simulate the tensile fracture of the rebar by finite element.

**Figure 24:**
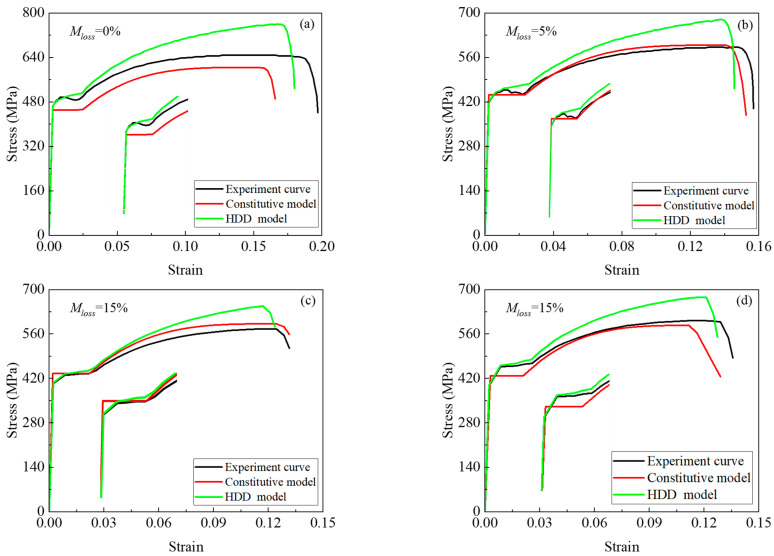
Comparative analysis of experimental data and constitutive equations: (**a**) *M_loss_* = 0%, stress and strain; (**b**) *M_loss_* = 5%, stress and strain; (**c**) *M_los_*_s_ = 10.5%, stress and strain; (**d**) *M_loss_* = 15%, stress and strain.

As shown in Figure 24a–d, rebar specimens 2new12, 2-4, 2-11, and 8-6 correspond to mass loss ratios of 0%, 5%, 10.5%, and 15%, respectively. The average stress–strain curves were obtained by dividing the applied load by the average corroded cross-sectional area, and the strain was calculated as the measured displacement divided by the original rebar length, based on the displacement–load relationships shown below. Using the experimentally derived stress–strain data, this study developed constitutive equations incorporating the mass loss ratios, the HDD model, numerical calculations, and software simulations. The simulated load–displacement curves showed good agreement with the experimental results. The simulated elastic modulus, yield strength, ultimate stress, and fracture strain were all within a reasonable error range (within 20%) compared to the experimental values. The linear degradation trend observed in the numerical simulations closely matched the experimental outcomes, with strength predictions aligning well with the test data. The constitutive model proposed in this study, based on mass loss ratios, can accurately predict the monotonic loading response of corroded steel reinforcement, providing a foundation for the subsequent nonlinear analysis of steel–concrete composite structures.

## 6. Conclusions

(1) When the mass loss ratios are within 16.28%, the stress–strain curves exhibit a distinct yield plateau, which becomes shorter as the degree of corrosion increases. The modulus of elasticity, yield strength, ultimate stress, and fracture strength of the corroded reinforcement do not decrease significantly, but the ductility decreases noticeably.

(2) Based on existing research results and considering the mechanical property degradation laws under different mass loss ratios, an intrinsic equation for corroded steel bars accounting for mass loss ratios is established.

(3) The ontological equation based on the mass loss ratios shows good agreement with both the experimental data and the HDD simulation model.

## Figures and Tables

**Table 1:** Chemical composition of HRB400 reinforcing steel.

Grade	Chemical Composition %					
	C	Si	Mn	P	S	Other
HRB400	0.25	0.8	1.6	0.045	0.045	0.54

**Table 2:** Dimensions of HRB400 reinforcing steel.

*d*	*d_l_*		*h*		*h*_1_: (≤)	*b*	*a*	*l*		Max End Clearance of Transverse Rib (10% of Nominal Perimeter)
	Nominal Size	Allowable Deviation	Nominal Size	Allowable Deviation				Nominal Size	Allowable Deviation	
12	11.5	±0.4	1.2	±0.1	1.6	0.7	1.5	8	±0.5	3.7

**Table 3:** Mechanical properties of the artificially corroded reinforcing bars.

Labels	*M_loss_* /%	*F_el_*/kN	*F_m_*/kN	*f*_y_/MPa	*f*_u_/MPa	*f_br_*/MPa	*f*_y_ */f*_u_	*k_y_*	*k_u_*	*δ* /%
1-1	6.89	43.41	60.83	420.33	589.01	516.58	0.71	0.87	0.91	0.13
1-3	8.51	43.72	60.51	423.33	585.91	555.17	0.72	0.87	0.90	0.15
1-4	−3.10	44.36	58.79	414.43	549.24	483.15	0.75	0.85	0.85	0.16
1-5	6.68	42.89	57.85	442.70	597.11	482.60	0.74	0.91	0.92	0.15
1-6	11.13	42.65	58.07	404.47	550.70	533.72	0.73	0.83	0.85	0.13
1-10	10.87	42.22	59.15	428.76	600.69	564.18	0.71	0.88	0.93	0.13
1-11	10.51	41.19	59.31	384.62	553.83	446.93	0.69	0.79	0.85	0.13
2-1	1.75	45.33	60.51	444.44	593.27	497.66	0.75	0.92	0.91	0.11
2-2	8.76	43.99	59.43	498.74	673.79	499.32	0.74	1.03	1.04	0.11
2-4	5.41	43.81	58.34	446.11	594.07	399.15	0.75	0.92	0.92	0.14
2-5	11.37	42.22	58.67	401.08	557.35	466.18	0.72	0.83	0.86	0.13
2-6	12.69	42.97	58.42	406.80	553.06	467.89	0.74	0.84	0.85	0.11
2-9	12.03	43.94	59.31	423.98	572.29	459.36	0.74	0.87	0.88	0.11
2-11	10.64	42.12	57.81	422.45	579.81	515.23	0.73	0.87	0.89	0.12
3-1	7.82	43.44	59.88	438.01	603.78	380.21	0.73	0.90	0.93	0.11
3-2	1.18	46.77	62.67	455.89	610.88	470.11	0.75	0.94	0.94	0.14
3-3	9.97	42.90	59.38	390.50	540.51	499.19	0.72	0.80	0.83	0.11
3-5	12.92	42.43	57.33	485.62	656.16	483.37	0.74	1.00	1.01	0.12
3-6	6.03	42.88	57.72	439.52	591.63	441.96	0.74	0.90	0.91	0.14
3-9	12.33	42.67	58.06	392.37	533.89	535.03	0.73	0.81	0.82	0.12
3-10	14.21	42.97	58.10	404.01	546.26	496.01	0.74	0.83	0.84	0.13
4-1	−2.30	42.22	60.94	397.54	573.80	457.71	0.69	0.82	0.88	0.13
4-2	10.45	41.79	58.89	439.17	618.87	354.39	0.71	0.90	0.95	0.12
4-3	12.19	42.47	58.72	456.20	630.75	496.32	0.72	0.94	0.97	0.12
4-4	11.45	42.61	59.50	414.75	579.16	481.24	0.72	0.85	0.89	0.13
4-5	5.90	43.21	57.93	442.30	592.97	487.59	0.75	0.91	0.91	0.13
4-9	12.86	40.40	58.35	369.61	533.84	542.52	0.69	0.76	0.82	0.12
4-11	12.31	41.71	59.24	429.70	610.29	385.06	0.70	0.88	0.94	0.13
5-2	11.94	41.70	57.74	428.82	593.77	489.31	0.72	0.88	0.92	0.09
5-3	12.82	44.20	59.30	488.37	655.21	497.64	0.75	1.01	1.01	0.11
5-4	12.98	42.86	57.69	444.74	598.62	456.72	0.74	0.92	0.92	0.12
5-5	5.13	42.08	57.49	427.24	583.70	502.56	0.73	0.88	0.90	0.13
5-6	16.28	41.37	56.37	472.59	643.95	508.36	0.73	0.97	0.99	0.11
5-9	13.08	41.85	56.39	471.80	635.72	461.89	0.74	0.97	0.98	0.13
5-11	3.23	45.15	60.44	449.43	601.63	523.52	0.75	0.93	0.93	0.14
6-1	6.87	45.32	60.71	470.26	629.96	469.73	0.75	0.97	0.97	0.13
6-2	10.29	44.02	59.83	443.07	602.20	466.36	0.74	0.91	0.93	0.13
6-4	4.79	44.09	59.19	446.07	598.83	483.06	0.74	0.92	0.92	0.13
6-5	13.69	42.44	57.47	441.17	597.42	438.34	0.74	0.91	0.92	0.13
6-11	3.32	44.98	60.65	448.15	604.28	478.76	0.74	0.92	0.93	0.13
7-2	11.47	41.63	60.22	376.39	544.46	474.42	0.69	0.77	0.84	0.13
7-4	2.60	45.15	60.62	446.51	599.51	479.85	0.74	0.92	0.92	0.15
7-5	5.41	43.30	58.46	440.94	595.32	463.74	0.74	0.91	0.92	0.15
7-6	15.08	42.59	57.93	409.53	557.04	447.90	0.74	0.84	0.86	0.14
7-9	11.47	42.87	58.17	380.51	516.32	493.89	0.74	0.78	0.80	0.10
7-10	11.01	41.83	58.33	403.63	562.84	483.92	0.72	0.83	0.87	0.11
7-11	13.60	45.59	61.15	508.27	681.75	480.22	0.75	1.05	1.05	0.11
8-1	6.19	48.94	65.21	455.43	606.84	530.18	0.75	0.94	0.94	0.12
8-2	4.56	44.06	59.70	444.70	602.56	514.76	0.74	0.92	0.93	0.15
8-3	11.00	46.31	62.94	485.78	660.23	444.12	0.74	1.00	1.02	0.10
8-4	10.91	47.26	63.01	455.23	606.94	451.92	0.75	0.94	0.94	0.13
8-5	4.10	46.08	61.26	462.86	615.34	452.64	0.75	0.95	0.95	0.11
8-6	14.67	45.45	60.27	416.52	552.34	484.37	0.75	0.86	0.85	0.12
8-11	11.52	42.78	58.30	432.13	588.90	463.49	0.73	0.89	0.91	0.11
9-1	11.56	43.51	60.01	398.74	549.95	478.12	0.73	0.82	0.85	0.12
9-2	10.57	43.34	59.51	435.45	597.92	500.28	0.73	0.90	0.92	0.11
9-4	10.88	46.62	59.11	472.60	599.21	473.80	0.79	0.97	0.92	0.13
9-5	4.29	44.10	59.33	443.85	597.14	447.24	0.74	0.91	0.92	0.15
9-6	15.20	42.27	57.96	426.22	584.42	511.32	0.73	0.88	0.90	0.12
9-9	1.34	47.84	62.63	467.06	611.45	480.50	0.76	0.96	0.94	0.12
9-11	11.05	44.36	59.39	446.50	597.78	485.76	0.75	0.92	0.92	0.08
1new12	0.00	49.98	65.37	482.00	630.12	534.96	0.76	1.00	1.00	0.15
2new12	0.00	50.12	66.00	484.00	640.34	414.07	0.76	1.00	1.00	0.15
3new12	0.00	50.42	67.36	485.67	648.84	515.03	0.75	1.00	1.00	0.15

**Table 4:** Partial parameters of constitutive model.

Labels	*M_loss_*/%	*ε_y_*/%	*ε_sh_*/%	*ε_u_*/%	*ε_br_*/%	*P*_1_	*P*_2_	*E*/GPa
1-1	6.89	0.23	3.444	15.497	16.789	4.021	4.579	183
1-3	8.51	0.23	2.15	14.187	15.906	3.13	7.123	184
1-4	−3.10	0.249	2.491	15.692	17.779	3.577	9.465	180
1-5	6.68	0.249	2.491	16.191	17.187	4.286	2.76	192
1-6	11.13	0.23	2.15	12.467	13.327	3.019	5.13	176
1-10	10.87	0.23	2.152	13.345	15.067	3.062	7.352	186
2-1	1.75	0.221	1.107	12.62	13.506	2.231	2.831	193
2-2	8.76	0.229	2.576	11.592	13.237	3.645	7.384	217
2-4	5.41	0.221	2.212	14.818	15.686	4.095	5.041	194
2-5	11.37	0.23	2.15	13.757	14.918	3.236	3.555	174
2-6	12.69	0.23	2.583	12.054	13.55	3.215	4.123	177
2-9	12.03	0.228	1.839	12.972	13.599	3.458	3.00	184
2-11	10.64	0.229	2.573	12.434	13.155	3.136	2.048	184
3-1	7.82	0.215	2.368	12.269	13.208	3.193	3.50	190
3-2	1.18	0.221	2.433	13.801	15.201	3.33	4.67	198
3-3	9.97	0.215	2.152	11.838	12.917	2.474	3.08	170
3-5	12.92	0.215	1.937	10.547	11.408	2.787	2.70	211
3-6	6.03	0.249	2.244	15.46	16.801	3.65	3.62	191
3-9	12.33	0.215	2.368	12.269	12.915	3.452	2.938	171
3-10	14.21	0.215	1.937	12.054	13.345	3.017	4.282	176
4-1	−2.30	0.221	2.657	14.613	15.477	3.676	2.512	173
4-2	10.45	0.215	1.935	10.748	11.516	2.814	2.27	191
4-3	12.19	0.215	0.861	11.193	12.053	2.103	2.70	198
4-4	11.45	0.231	2.154	14.655	15.316	3.732	1.75	180
4-5	5.90	0.222	2.66	14.629	15.516	3.525	2.669	192
4-9	12.86	0.215	1.932	12.236	12.88	3.278	3.45	161
4-11	12.31	0.203	2.015	12.494	13.431	3.33	3.402	187
5-2	11.94	0.215	2.152	10.116	10.898	3.18	2.615	186
5-3	12.82	0.215	2.34	11.153	12.227	3.878	3.46	212
5-4	12.98	0.215	2.152	11.838	12.915	4.345	2.75	193
5-5	5.13	0.221	2.435	13.949	14.613	3.239	2.655	186
5-6	16.28	0.215	2.152	11.408	12.072	3.144	2.546	205
5-9	13.08	0.203	2.015	11.285	13.063	3.056	7.36	205
5-11	3.23	0.249	2.488	14.182	15.494	3.934	6.531	195
6-1	6.87	0.203	2.015	12.494	13.921	3.053	3.732	204
6-2	10.29	0.203	1.854	12.736	14.159	3.435	5.00	193
6-4	4.79	0.234	1.854	14.151	14.853	3.447	1.95	194
6-5	13.69	0.204	2.021	12.932	14.721	3.713	5.931	192
6-11	3.32	0.221	2.878	14.392	15.277	3.234	2.50	195
7-2	11.47	0.202	2.415	12.88	13.685	4.255	2.484	164
7-4	2.60	0.249	3.629	16.568	17.984	3.507	3.94	194
7-5	5.41	0.249	2.244	15.46	16.707	3.449	3.576	192
7-6	15.08	0.204	2.018	13.318	14.643	4.034	4.03	178
7-9	11.47	0.215	2.15	11.178	11.772	3.227	2.116	165
7-10	11.01	0.215	2.15	10.748	11.607	3.167	2.923	175
7-11	13.6	0.215	2.15	10.103	11.393	3.02	4.131	221
8-2	4.56	0.249	2.491	15.568	17.311	3.695	4.911	198
8-3	11.00	0.23	2.15	11.178	12.301	3.31	3.47	193
8-4	10.91	0.23	2.152	12.484	13.718	3.145	3.30	211
8-5	4.10	0.221	2.657	13.063	14.018	3.429	2.50	201
8-6	14.67	0.23	2.579	12.037	13.577	3.184	4.871	181
8-11	11.52	0.215	1.932	10.089	11.141	2.679	2.98	188
9-1	11.56	0.215	1.932	11.807	12.807	3.123	3.05	173
9-2	10.57	0.215	2.361	12.236	13.095	3.257	2.57	189
9-5	4.29	0.249	2.491	15.692	17.934	3.999	4.73	193
9-6	15.2	0.23	2.583	13.345	14.206	4.006	3.672	185
9-9	1.34	0.194	2.133	11.055	11.831	2.69	2.00	203
9-11	11.05	0.215	2.152	9.040	9.901	2.771	2.386	194
1new12	0.00	0.249	2.485	16.043	17.895	4.00	4.667	210
2new12	0.00	0.276	2.212	16.587	19.708	4.161	6.65	210
3new12	0.00	0.277	1.939	14.962	16.937	3.309	6.54	211

**Table 5:** Mechanical properties of HRB400 rebar.

Density, *ρ* (kg/m^3^)	7800
Young’s modulus, *E* (GPa)	210
Poisson’s ratios, *ν*	0.28
Yield strength, *σ*_y0_ (MPa)	440
Tensile strength, *σ*_u_ (MPa)	560
Fracture strain, *e*_u_ (%)	25
Failure displacement (mm)	0.14

**Table 6:** Parameters of the constitutive equations for different mass loss ratios of some steel bars.

Labels	*M_loss_*/%	*E*/GPa	ε*_y_*/%	ε*_sh_*/%	ε*_u_*/%	ε*_br_*/%	*P*_1_	*P*_2_	*f_y_*	*f_u_*	*f_br_*
2new12	0	195.77	0.24	2.45	15.14	16.62	3.52	4.59	604	450	478
2-4	5	194.62	0.2305	2.33	13.89	15.22	3.435	3.39	599	442	399
2-11	10.5	193	0.22005	2.198	12.515	13.68	3.3415	2.07	592	435	477
8-6	15	192.32	0.2115	2.09	11.39	12.42	3.265	0.99	587	428	476

## Data Availability

The original contributions presented in this study are included in the article. Further inquiries can be directed to the corresponding author.
